# MicroRNA-223 and microRNA-21 in peripheral blood B cells associated with progression of primary biliary cholangitis patients

**DOI:** 10.1371/journal.pone.0184292

**Published:** 2017-09-08

**Authors:** Xiaomei Wang, Xiaoyu Wen, Jingjing Zhou, Yue Qi, Ruihong Wu, Yao Wang, Yiwen Kui, Rui Hua, Qinglong Jin

**Affiliations:** 1 Department of Hepatology, The First Hospital of Jilin University, Changchun, China; 2 Key Laboratory of Zoonosis Research, Ministry Education, Changchun, China; Emory University School of Medicine, UNITED STATES

## Abstract

Recently, there is ample evidence suggesting the important role of microRNAs (miRNAs) in autoimmune diseases via modulating B cells function. Primary biliary cholangitis (PBC) is a progressive immune-mediated liver disease with unclear pathogenic mechanism. Whether the miRNA in peripheral B cells of PBC involve the mechanisms of pathology and progression is not known. The present study explores miRNA deregulation in peripheral B-cell of PBC from stage I to IV and healthy controls. Peripheral B cells were obtained from 72 PBC patients (stage I, n = 17; stage II, n = 23; stage III, n = 16; stage IV, n = 16) and 15 healthy controls. Initially, in a discovery study, miRNA array analysis was performed, subsequently, in a validation study, quantitative PCR was used to investigate miRNA expression profile in B cells of PBS patients compared to healthy controls. Based on bioinformatics analysis, we identified the potential biological processes and significant signaling pathways affected by these microRNAs, and generated the microRNA–gene network. The discovery study identified 558 miRNAs differentially expressed in B cells of PBC patients compared to controls. Interestingly, among all differentially expressed miRNAs, hsa-miR-223-3p and hsa-miR-21-5p were the only miRNAs that showed consistent and significant down-regulation from stage I to stage III of PBC. Bioinformatics showed that potential target genes of both miRNAs involved in migration, cell differentiation, apoptosis, and signal transduction pathways. In conclusion, our results suggest that the expression profiles of miRNA in peripheral B cells of PBC patients are closely associated with the disease progression, especially the down-regulation of hsa-miR-223-3p and hsa-miR-21-5p. Taken together, our study offers novel perspectives on the role of miRNAs in PBC pathogenesis.

## Introduction

Primary biliary cholangitis (PBC) is a progressive immune-mediated liver disease characterized by damage of the small intrahepatic bile ducts, resulting in periportal inflammation, fibrosis and cirrhosis. The disease mainly affects mostly middle-aged women [1]. Etiologically, PBC is believed to result from a combination of a genetic and environmental factors[2]. The treatment of PBC is ursodeoxycholic acid(UDCA) in the past 30 years and obeticholic acid(OCA) approved in May 2017 by FDA, which increases the transplant-free survival, especially when starts early in the course of disease[3]. However, a substantial proportion (about 40%) of PBC patients does not respond to ursodeoxycholic acid treatment. Therefore, identifying novel cellular molecules and mechanisms contributing to PBC pathogenesis are needed to open new insights for novel treatments.

microRNAs (miRNAs) are small noncoding RNA (19 to 24 nucleotides) that can bind to complementary sites on the target mRNA and hence alter its transcription and translation [4–6]. Currently, more than 2500 mature human miRNAs are listed in the miRBase database[7]. miRNAs affect various important cell functions such as cell division, differentiation, apoptosis, carcinogenesis, and immune function [8]. Different pathological conditions including autoimmune diseases, cancers, and liver diseases have different miRNA expression profiles [9–11]. Furthermore, evidence is growing that miRNA expression profiles are different among different liver diseases and some microRNA may relate to the progression stage of liver disease[12].

Although some studies focused on global miRNA expression profiles either in liver biopsies or peripheral blood mononuclear cells (PBMC) of PBC patients [13, 14], the molecular mechanism and etiology of this enigmatic autoimmune disease is still not fully known. The transition from innate immunity to acquired immunity is followed by the presence of autoreactive T and B cells, which are involved in high titer serum anti-mitochondrial antibodies (AMA) and an autoimmune-mediated destruction of small intrahepatic bile ducts [15]. There have been no prior studies of miRNA expression patterns in B cells from different pathological stages of PBC patients. In the current study we investigated for the first time the expression profile of miRNAs in B cells, producer of AMA, of PBMC obtained from Chinese patients with different pathological stages of PBC. This approach might reveal novel mechanistic insights of miRNAs role in PBC pathogenesis and identifying their gene targets.

## Materials and methods

### PBC patients and healthy controls

The study involved 87 subjects (72 PBC patients and 15 healthy controls) recruited from The First Hospital, Jilin University (Changchun, China) between 2014 and 2016. The PBC patients were diagnosed according to the AASLD Practice Guideline[16]. The diagnosis of cirrhosis was based on liver biopsy and all patients were treatment naive. Exclusion criteria included individuals infected with HBV, HCV and HDV, or other inflammatory diseases, patients presented with rheumatoid arthritis, diabetes, autoimmune hepatitis, hypertension. The healthy controls had no medical history of any liver disease, autoimmune diseases or other diseases. The experimental protocol was approved by the Ethics Committee of the First Hospital of Jilin University. An informed written consent was obtained from each patient.

Based on histologic lesions, PBC progression was divided into four stages. Stage I is characterized by portal inflammation with or without bile duct involvement. Stage II is characterized by the gradual increase of periportal lesions extending into the hepatic parenchyma. Stage III is characterized by a alteration of the hepatic architecture with numerous fibrous septa. Cirrhosis with the existence of regenerative nodules defines stage IV[17]. Accordingly, among the 72 PBC patients involved in this study, there were 17, 23, 16 and 16 patients with stages I, II, III and IV, respectively. The healthy controls were age and gender matched with the PBC patients. Detailed clinical information is shown in Table 1.

**Table 1:** Clinical characteristics of PBC patients and healthy controls.

	Stage I (n = 17)	Stage II (n = 23)	Stage III (n = 16)	Stage IV (n = 16)	Control (n = 15)	*P* value
Gender (Female/male)	16/1	21/2	16/0	16/0	13/2	
Age	53.1±3.8	54.8±4.9	51.7±3.4	52.7±5.6	48.0±4.5	0.11
AST	72.9±18.7	78.6±11.4	83.3±24.4	97.6±24.4	26.0±2.9	0.24
ALT	69.2±26.1	88.8±12.2	83.4±12.2	84.2±30.7	27.0±5.5	0.46
GGT	344.7±104.5	445.±102.4	460.6±144.0	466.1±139.1	25.0±3.4	0.16
ALP	290.2±73.2	300.7±44.1	358.2±142.6	212.1±65.6	56.3±11.8	0.20
ALB	39.1±1.6	37.7±1.0	35.7±1.0	27.8±3.8	47.0±3.2	0.001
TB	16.5.±5.6	21.1.±6.9	51.5.±8.7	61.3.±10.4	15.4.±3.2	0.024

AST, aspartate transaminase; ALT, alanine aminotransferase; GGT, gamma- glutamyl- transpeptidase; ALP, alkaline phosphatase; ALB, albumin; TB, total bilirubin. PBC, primary biliary cholangitis.NA, not available.

### B cell collection and RNA isolation

Venous blood samples (20 ml) were collected in EDTA-treated tubes from all subjects. Next, we applied density gradient centrifugation (Histopaque^®^-1077, Sigma-Aldrich, Saint Louis, USA) to isolate the PBMCs. B cells were separated from PBMCs using CD19 microbeads (Miltenyi, Cologne, Germany). Purity of the CD19+ B cells population was assessed by fluorescence-activated cell sorting (FACS) and was found to be 92%–97%. Total RNA was harvested from B cells using TRIzol (Invitrogen, Carlsbad, USA) according to the manufacturer’s instructions. In discovery study, a total of 39 RNA samples obtained from 32 PBC patients and 7 healthy controls were used for miRNA array analysis, while in validation study, additional 48 RNA samples obtained from 40 PBC and 8 healthy controls were used.

### MicroRNA array analysis

The RNA samples from B cells isolated from PBC patients or healthy control were labeled using a miRCURY^™^ Array Power Labeling kit (Exiqon, Vedbaek, Denmark). Next, the labeled RNA was hybridized on a miRCURY^™^ LNA Array (v.18.0, Exiqon). Array slides were scanned using an Axon GenePix 4000B Microarray Scanner (Axon Instruments, Foster, USA) Data were extracted from scanned images using GenePix Pro 6.0 (Axon Instruments). All the microarray analysis was performed by the Exiqon miRNA Expression Profiling service (Kangchen Bio-tech, China).

### Quantitative real-time polymerase chain reaction (qPCR)

We used qPCR to validate the differentially expressed miRNAs identified in the validation study of miRNA arrays. Total RNA extracted from PBMCs was used to synthesize cDNA using miRNA-specific, stem-loop reverse transcription (RT) primers and reagents from a MMLV Reverse Transcriptase kit (Epicentre, Madison, USA), following the manufacturer’s protocol. Next, we performed qPCR with ViiA 7 Real-time PCR System (Applied Biosystems, Foster, USA), and 2×SYBR-Green PCR master mix (Arraystar, Rockville, USA) according to the manufacturer’s instructions. We used the following primers: hsa-miR-223-3p F: 5’GGGGTGTCAGTTTGTCAAA3’, R:5’CAGTGCGTGTCGTGGAGT3’. hsa-miR-21-5p F: 5' GGGGGGTAGCTTATC AGACTG3', R:5'CAGTGC GTGTCGTGGAGT3'. All reactions were performed in triplicate. The expression level of each miRNA was calculated relative to U6 RNA. The relative expression level of each miRNA was calculated using the equation 2^-ΔΔCt^.

### Bioinformatic analysis of differentially expressed miRNAs

To evaluate functions of candidate miRNAs of PBC patients, target genes prediction were performed using the TargetScan database (http://www.targetscan.org/vert_70/)[18].

The functional annotation of target genes were performed using the DAVID,v6.7 (https://david.ncifcrf.gov/summary.jsp) [19]. DAVID provides functional annotation of a gene list based on gene ontology (GO) terms, the Kyoto Encyclopedia of Genes and Genomes (KEGG) pathway as well as the BioCarta pathway databases. [13].

### Statistical analysis

For the discovery study, the non-normally distributed continuous variables of differentially expressed miRNAs between cases and controls were compared by Mann- Whitney U test. Different miRNAs were compared using significant analysis and false discovery rate (FDR) analysis. the FDR was calculated to correct the p-value. FDR≤0.2 indicated smaller errors in judging p-values.

For the validation study, one-way ANOVA was applied to extract differentially expressed miRNAs in qPCR among the four groups of PBC and controls. P-values of less than 0.05 were considered statistically significant.

## Results

### Patient population

Demographic characteristics and blood biochemical parameters of PBC patients with different 4 stages of the disease as well as healthy controls were summarized in Table 1.

### miRNA expression profile of B cell in PBC and controls

Expression profiles of miRNAs obtained from blood B cell of PBC patient and controls were compared using miRNA arrays. After normalization of the raw data and random variance model, we found a total of 558 miRNAs that differentially expressed between the PBC patients and controls. In particular, this analysis identified 266 up-regulated microRNAs (>2-fold) and 292 down-regulated microRNAs (<0.5-fold) in PBC patients compared to controls.

Moreover, we found differentially expressed miRNAs even among the four stages of PBC, suggesting the association between miRNA expression profile and the progression stage of the disease. (Table 2). Notably, more advanced disease-stage showed more differentially expressed miRNA compared to less advanced disease-stage.

**Table 2:** Number of differentially expressed miRNAs among groups.

Compare		up	down	Total
Control vs	Stage I	212	231	443
	Stage II	285	300	585
	Stage III	236	292	528
	Stage IV	241	288	529
Stage IV vs	Stage I	77	110	187
	Stage II	0	14	14
	Stage III	2	8	10
Stage III vs	Stage I	62	92	154
	Stage II	1	10	11
Stage II vs	Stage I	131	104	235

### Differential expression of miRNAs in B cells from four PBC stages

Among all 558 differentially expressed miRNAs identified in discovery study, hsa-miR-223-3p and hsa-miR-21-5p were the only miRNAs showed consistent and significant down-regulation from stage I to stage III, (Fold Change<0.5, p<0.05), while the expression levels of both miRNAs were not different between stages III and V (Table 3). This indicates that the more advanced PBC-stage the more inhibition of hsa-miR-223-3p and hsa-miR-21-5p expression.

**Table 3:** Expressed of miR-223-3p and hsa-miR-21-5p in peripheral blood B cells of PBC patients and controls.

miRNA	Control Mean±SE	Stage I Mean±SE	Stage II Mean±SE	Stage III Mean±SE	Stage IV Mean±SE	Expression	p value
hsa-miR-223-3p	44.62±11.65	16.31±3.68	7.05±1.97	2.37±1.12	1.62±0.89	down	<0.001
hsa-miR-21-5p	52.45 ±2.80	17.20±2.59	6.46±1.04	2.03±0.25	1.86±0.33	down	<0.001

### Validation of miRNA array results by qPCR

**Fig 1:**
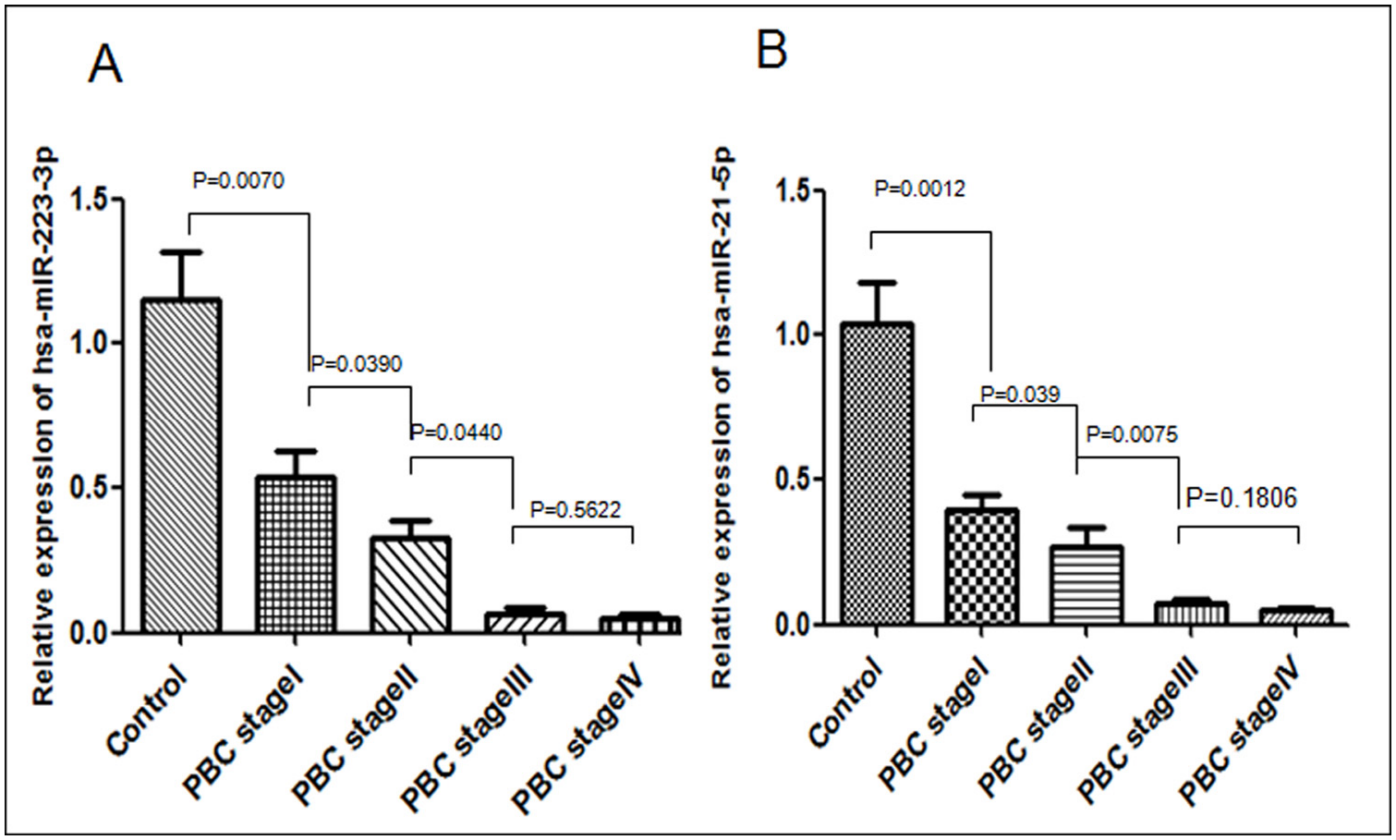
qPCR validation of peripheral expression of two miRNAs. [A] The expression of miR-223-3P in four stages of PBC patients and controls. [B] The expression of miR-21-5P in four stages of PBC patients and controls.

To validate the differential expression of hsa-miR-223-3p and hsa-miR-21-5p observed in discovery study of miRNA array analysis, qPCR were performed as a confirmatory method. Validation of the miRNA results was conducted in additional 48 samples (40 PBC patients and 8 healthy controls), which are different from the initial 39 RNA samples used in discovery study. Indeed, the relative expression of hsa-miR-223-3p, hsa-miR-21-5p was significantly different between PBC patients and controls. Furthermore, the expression of both miRNA was also different among stages I, II and III of PBC stages, confirming the results obtained from miRNA array analysis of discovery study (Fig 1).

### Microarray-based GO analysis and pathway analysis

We predicted the target genes of hsa-miR-223-3p and hsa-miR-21-5p using online search algorithms for target prediction (TargetScan, http://www.targetscan.org). There are 412 target genes for hsa-miR-223-3p and 382 target genes for hsa-miR-21-5p. Number of B cell-related predicted target genes of hsa-miR-223-3p and hsa-miR-21-5p were 10, 12, respectively. Target genes were annotated using DAVID v6.7 (https://david.ncifcrf.gov/summary.jsp).

For these putative target genes, GO analysis and KEGG pathway analysis were applied to display the miRNA–gene regulatory network. The most significant GO categories for the target genes of hsa-miR-21-5p and hsa-miR-223-3p are regulation of macromolecule metabolic process, regulation of transcription and regulation of cell migration (Table 4). Results in GO biological process(BP) indicated that6 functions matched ranking score ≥2.0 were identified in PBC.

**Table 4:** Top 10 significant GO categories for hsa-miR-223-3p and hsa-miR-21-5p target genes.

Term ID	Term	Count	%	P value
GO:0010604	positive regulation of macromolecule metabolic process	73	10.33994	5.95E-09
GO:0045449	regulation of transcription	161	22.80453	3.53E-08
GO:0030334	regulation of cell migration	25	3.541076	1.26E-07
GO:0009891	positive regulation of biosynthetic process	59	8.356941	2.59E-07
GO:0010628	positive regulation of gene expression	52	7.365439	3.13E-07
GO:0040012	regulation of locomotion	26	3.68272	3.78E-07
GO:0051270	regulation of cell motion	26	3.68272	4.17E-07
GO:0031328	positive regulation of cellular biosynthetic process	57	8.073654	8.08E-07
GO:0010629	negative regulation of gene expression	45	6.373938	2.42E-06
GO:0007423	sensory organ development	27	3.824363	3.11E-06

**Fig 2:**
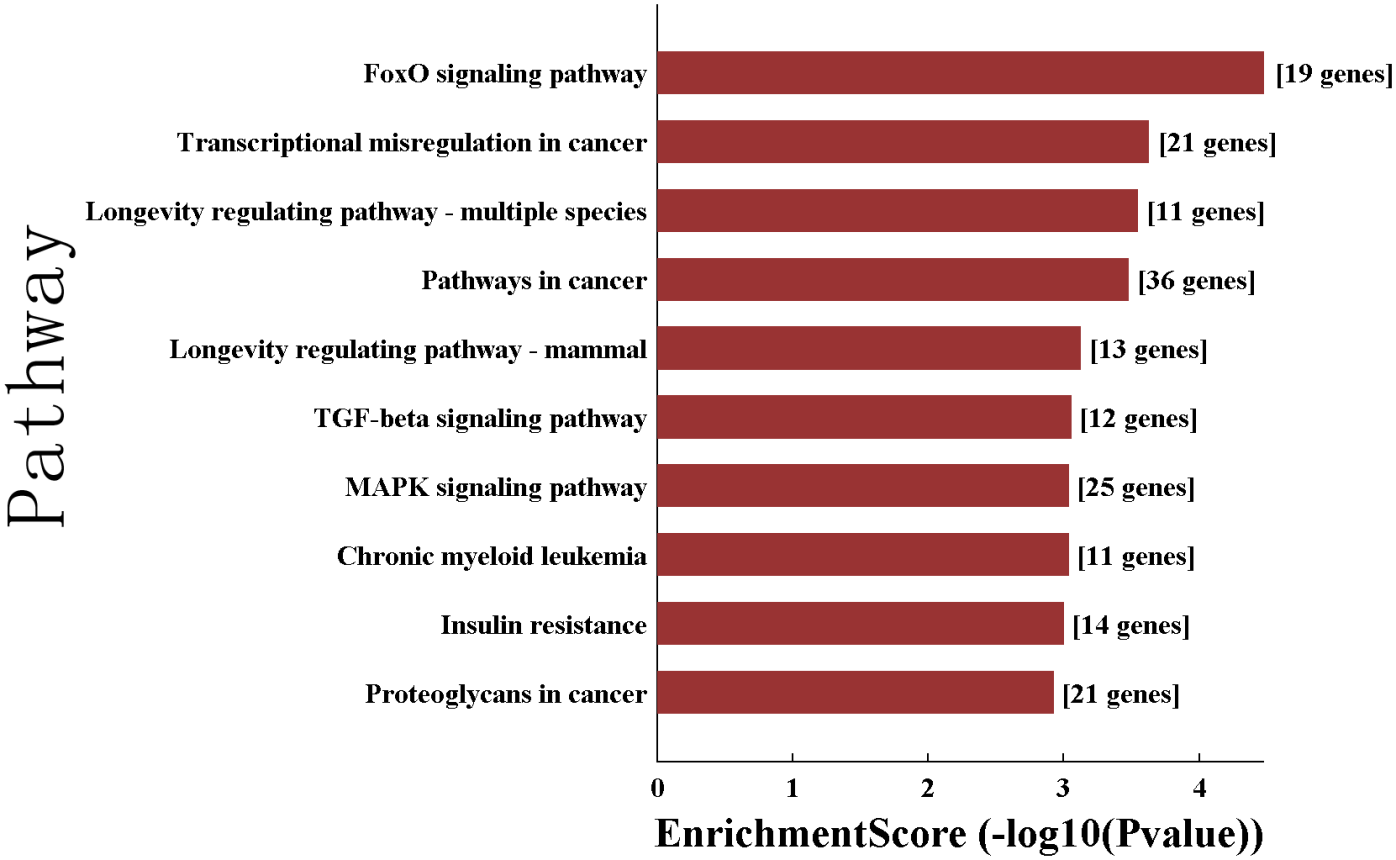
Pathway analysis based on potential target genes. The 10 top canonical KEGG pathways targeted by hsa-miR-223-3p, hsa-miR-21-5p are listed.

Also, these predicted target genes were entered in gene set enrichment analysis (GSEA) using KEGG database. The top 10 canonical KEGG pathways were shown in Fig 2, including FoxO, TGFβ and MAPK signaling pathways, and transcriptional misregulation in cancer.

### Identification of microRNA–B-cell related gene network

**Fig 3:**
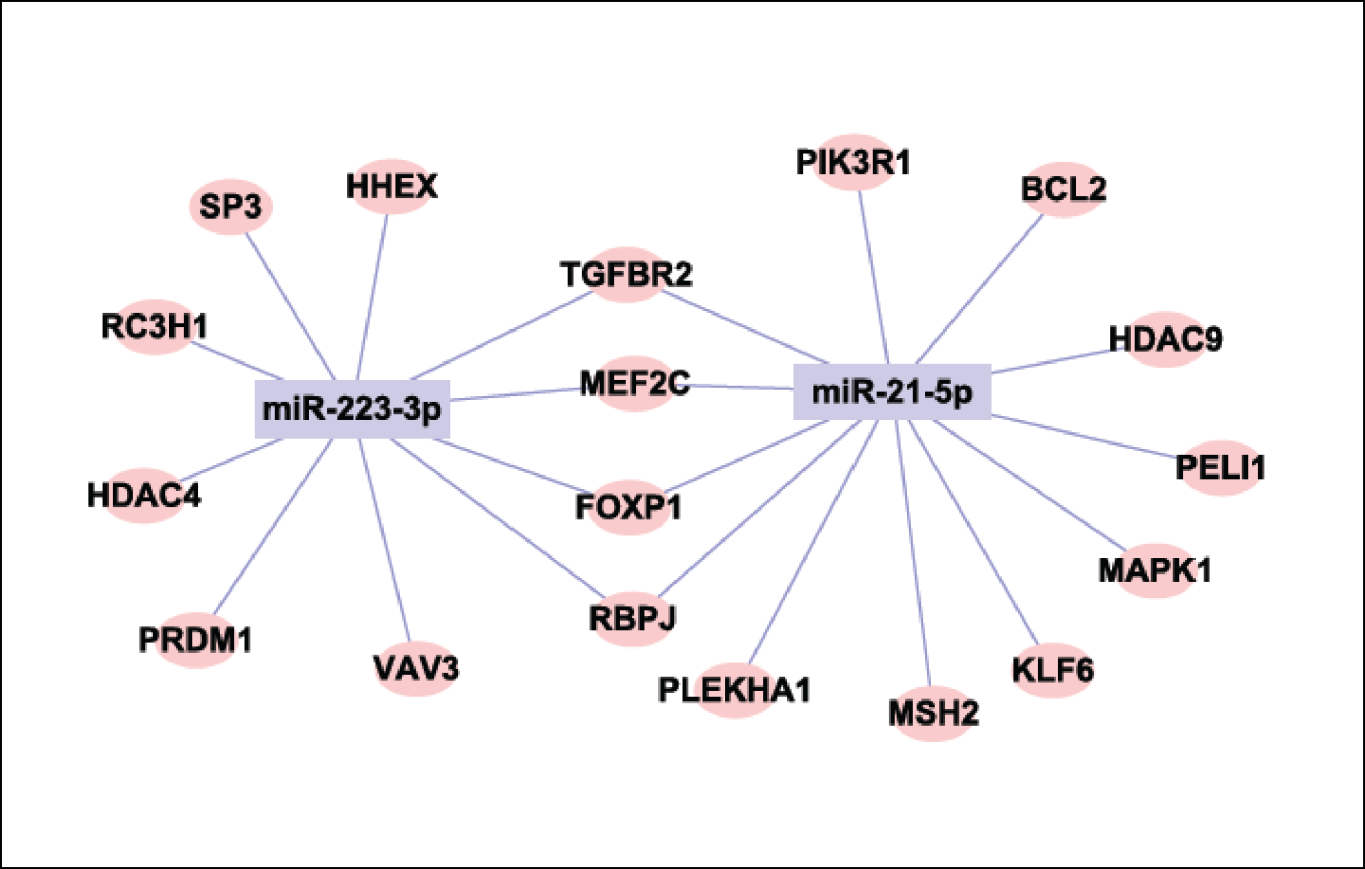
MicroRNA-target gene networks of miR-223-3P, miR-21-5P. The target genes related to functions of B-cell. The pink ellipse represents gene (mRNA); gray square represents microRNA. The relationship between microRNA and gene is represented by gray line.

Based on the putative target B cell function related genes of hsa-miR-21-5p and hsa-miR-223-3p, the microRNA gene regulatory network was constructed to outline the interactions of both microRNAs and their targets. As shown in Fig 3, two microRNAs (miR-223-5p, miR-21-5p) were in the center of the microRNA–gene network. There were mutual 4 target genes related B cell function of miR-223-5p, miR-21-5p, they were TGFBR2, MEF2C, FOXP1 and RBPJ. These target genes are related to B cell function, such as B-cell differentiation, activation, receptor signaling pathways, and homeostasis.

## Discussion

Recently, there is ample evidence suggesting the important role of miRNAs in autoimmune diseases[20, 21]. Since PBC is a model of autoimmune disease, but the pathogenic mechanism is not been full understood. Therefore, in the current study we aimed to investigate the association between miRNAs expression profile in peripheral B cells and the progression of PBC. B cells contributed in initiating liver inflammation, cyst formation and salivary gland pathology in a PBC mouse model, illustrating a critical role for B cells in modulating specific organ pathology [22].

Both discovery and validation studies of current investigation confirmed the significant association of the expression profile of the two miRNAs (hsa-miR-223-3p and hsa-miR-21-5p) with PBC. More importantly, the expression levels of hsa-miR-223-3p and hsa-miR-21-5p were not only associated with PBC compared to healthy controls, but they were also significantly correlated to the progression-stage of PBC. The expression levels of both miRNA were decreased significantly with progression from early to advanced clinical stages.

In chronic lymphoid leukemia (CLL), the expression of hsa-miR-223-3p in B cells significantly decreases with the progression from early to advanced clinical stages [23]. Our observations and Zhou et al., results suggest that hsa-miR-223-3p might play an important role in the pathogenesis of B cell-related diseases [23]. Additionally, hsa-miR-223-3p was implicated in the autoimmune disease ulcerative colitis [24]. Taken together, hsa-miR-223-3p can be involved in the pathogenesis and progression of autoimmune diseases, including PBC. Future in vivo and in vitro mechanistic studies are needed to elucidate this issue

Hsa-miR-21-5p is associated with tumors in several organs [25] and several liver diseases including nonalcoholic steatohepatitis [26], drug-induced liver injury [27], and hepatocellular carcinoma [28, 29]. However, the relationship between hsa-miR-21-5p and autoimmune disease has not been elucidated yet. Thus, our study is the first to suggest a possible link between hsa-miR-21-5p and PBC. Also, future in vivo and in vitro mechanistic studies are needed to elucidate the role of hsa-miR-21-5p in autoimmune diseases, in particular PBC.

In this study, GO analysis was used to obtain insights into the molecular function and biological processes of our identified target genes of hsa-miR-223-3p and hsa-miR-21-5p. This analysis highlighted the possible role of both miRNA in modulating B cell functions, including biological processes regulated by the two differentially expressed miRNAs included B-cell signal transduction, cell differentiation, cell migration, and apoptosis in GO categories

On the other hand, KEGG pathways analysis was performed to further investigate the relationship between the two downregulated miRNAs and PBC pathogenesis. Several signaling pathways previously proposed to impact PBC pathogenesis were also identified as top candidates in our study such as. TGF-β1 signaling, and MAPK signaling[30–32].. Again, further studies are needed to determine how the down-regulation of hsa-miR-223-3p and hsa-miR-21-5p influence the progression of PBC.

This study identified 4 mutual target genes (TGFBR2, MEF2C, FOXP1 and RBPJ) related B cell function of miR-223-5p and miR-21-5p. Notably,TGFBR1 is a critical member in TGF-β1 signaling way. Interestingly, dominant negative TGFBR2 mice has been verified would develop serological and histological features resembling human primary biliary cholangitis[33, 34]. This suggests that miR-223-5p and miR-21-5p might regulate the progression of PBC via TGF-β1 signaling pathway in TGFBR2-dependent manner.

In conclusion, our results indicate that B cells from patients with various stages of PBC have different miRNA expression profiles. In particular, the down-regulation of hsa-miR-223-3p and hsa-miR-21-5p during the course of PBC progression would be a useful clinical biomarker. Taken together, our study offers novel perspectives on role of microRNAs in PBC pathogenesis, however, the precise mechanism needs further experimental investigation.

## Supporting information

S1 Table Differentially expressed miRNAs. (XLSX)

S1 Fig Heat map and hierarchical clustering of PBC vs. Control. (TIF)

S2 Fig Amplification plot of hsa-miR-21-5p. (TIF)

S3 Fig Amplification plot of hsa-miR-223-3p. (TIF)
