# Supplementary figures and images for: MicroRNA-223 and microRNA-21 in peripheral blood B cells associated with progression of primary biliary cholangitis patients
Source: PLoS One. 2017 Sep 8;12(9):e0184292. doi: 10.1371/journal.pone.0184292 (PMC5590910; doi:10.1371/journal.pone.0184292)

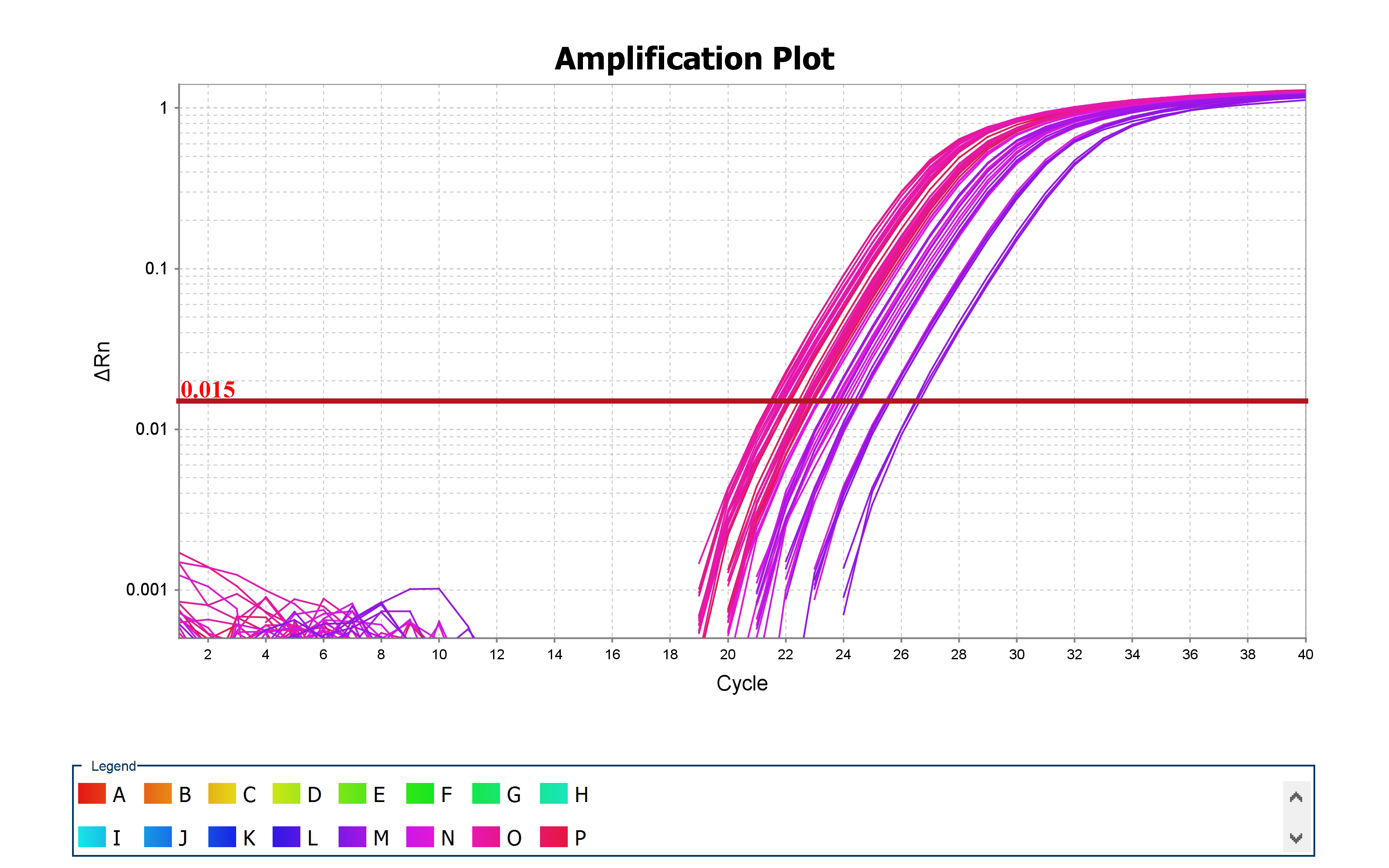

Supplement: S2 Fig — (TIF) [file pone.0184292.s003.tif]

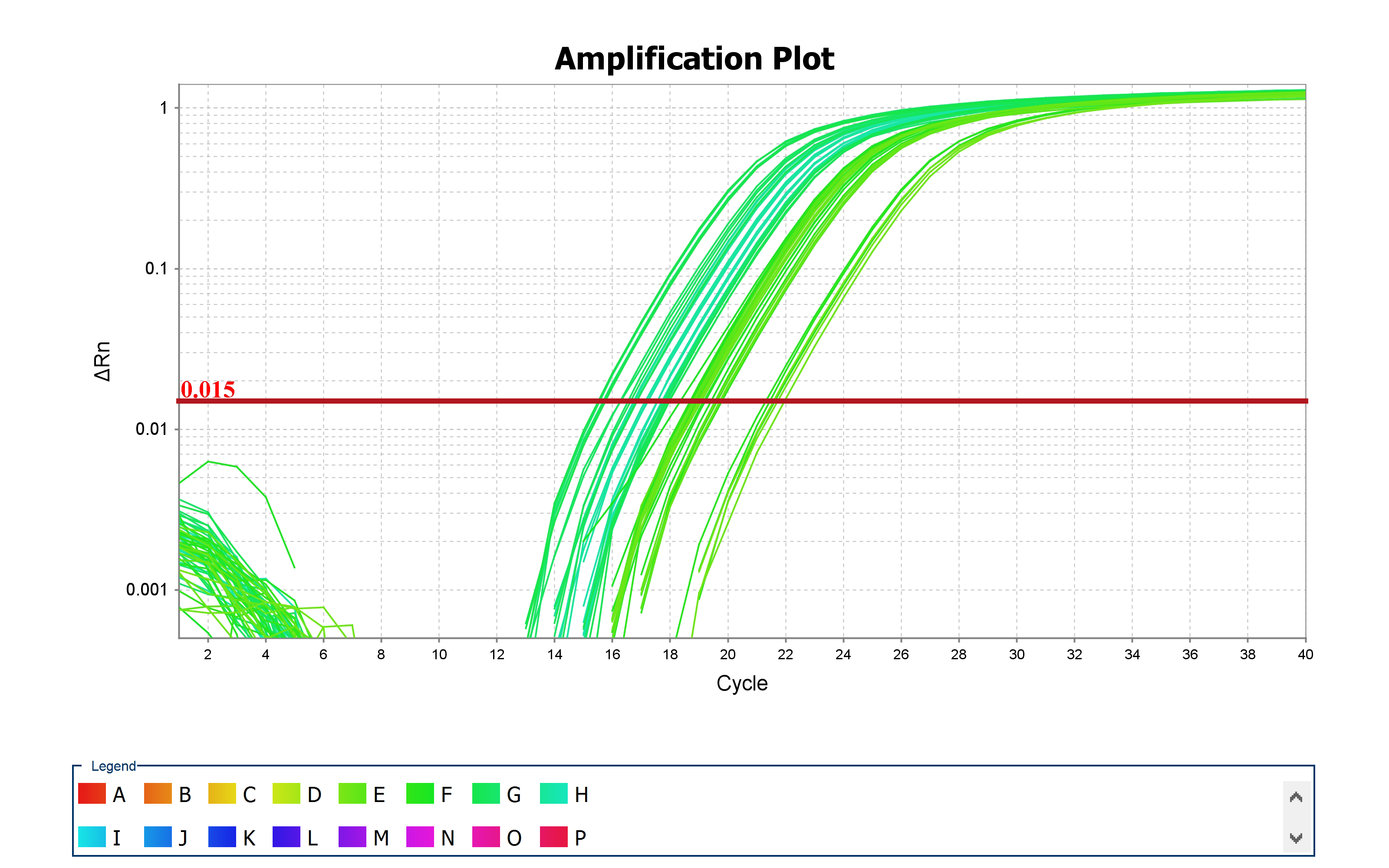

Supplement: S3 Fig — (TIF) [file pone.0184292.s004.tif]
